# Visualising household air pollution: Colorimetric sensor arrays for monitoring volatile organic compounds indoors

**DOI:** 10.1371/journal.pone.0258281

**Published:** 2021-10-06

**Authors:** Emer Duffy, Kati Huttunen, Roosa Lahnavik, Alan F. Smeaton, Aoife Morrin

**Affiliations:** 1 Insight SFI Research Centre for Data Analytics, Dublin City University, Dublin, Ireland; 2 National Centre for Sensor Research, School of Chemical Sciences, Dublin City University, Dublin, Ireland; 3 Department of Environmental and Biological Sciences, University of Eastern Finland, Kuopio, Finland; The University of Alabama, UNITED STATES

## Abstract

Indoor air quality monitoring as it relates to the domestic setting is an integral part of human exposure monitoring and health risk assessment. Hence there is a great need for easy to use, fast and economical indoor air quality sensors to monitor the volatile organic compound composition of the air which is known to be significantly perturbed by the various source emissions from activities in the home. To meet this need, paper-based colorimetric sensor arrays were deployed as volatile organic compound detectors in a field study aiming to understand which activities elicit responses from these sensor arrays in household settings. The sensor array itself is composed of pH indicators and aniline dyes that enable molecular recognition of carboxylic acids, amines and carbonyl-containing compounds. The sensor arrays were initially deployed in different rooms in a single household having different occupant activity types and levels. Sensor responses were shown to differ for different room settings on the basis of occupancy levels and the nature of the room emission sources. Sensor responses relating to specific activities such as cooking, cleaning, office work, etc were noted in the temporal response. Subsequently, the colorimetric sensor arrays were deployed in a broader study across 9 different households and, using multivariate analysis, the sensor responses were shown to correlate strongly with household occupant activity and year of house build. Overall, this study demonstrates the significant potential for this type of simple approach to indoor air pollution monitoring in residential environments.

## Introduction

Over 2 million healthy life years are lost annually across the European Union as a result of indoor air pollution [1]. In developed countries we spend 80–90% of our time indoors, where our exposure to air pollution can be substantially greater than exposures occurring outdoors. The majority of this time indoors is spent in the home, therefore, individual exposure to air pollution is strongly influenced by household conditions and activities [2]. The concentrations of many air pollutants can be higher in homes than elsewhere, particularly following activities like cooking and cleaning.

Volatile organic compounds (VOCs) are a diverse group of chemical compounds present in indoor air that frequently reach higher indoor concentrations compared to outdoors. The primary sources of VOCs in indoor environments include outdoor air (penetration from outdoors to indoors) and indoor sources such as building materials and furnishings [3], consumer products [4], occupants [5] and occupant activities such as cooking and cleaning [6–8]. Some of the most commonly found VOCs are BTXS (benzene, toluene, xylenes and styrene), terpenes (limonene, μ-pinene) and carbonyl compounds (acrolein, formaldehyde) [9]. Chemical reactions occurring in the indoor environment are a secondary source of indoor air pollution. Unsaturated organic compounds can react with oxidants to produce additional pollutants, such as the oxidation of terpenes to produce carbonyl compounds (*e*.*g*. formaldehyde, acetaldehyde, and other C_5_-_10_ aldehydes).

Measurement of indoor air pollution is an integral part of exposure monitoring and human health risk assessment. In-field monitoring of VOCs should be based on a fast, economical and easy-to-use detection system to elucidate the roles of various pollution sources (*e*.*g*. building materials or occupant activities like cooking, cleaning, *etc*.). Traditional measurement approaches for VOC monitoring indoors are generally reliant on high-end instrumentation including PTR-TOF-MS [5, 8]. Field surveys of multiple buildings typically rely on smaller and less expensive instrumentation for measurement of a limited set of analytes, or on deployment of passive samplers followed by off-line analysis [10]. These approaches provide high quality data, but their high cost and low deployment density limit the scale of air pollution studies. Low-cost sensors can enhance indoor air pollution monitoring capabilities by enabling higher deployment density and collection of more representative exposure data in a variety of settings. Various sensors have been proposed for VOC monitoring including electrochemical resistors [11], quartz crystal microbalances [12] and colorimetric sensor arrays (CSAs) [13–15].

Colorimetric detection of VOCs has received much attention due to its simplicity, cost-effectiveness and ease of visualisation. It has shown a strong ability to detect diverse VOCs in air samples [16–20], making it a promising approach for monitoring VOC air pollution indoors. Colorimetric sensors detect the change in colour of a pigment upon interaction with analyte vapours, an approach that is based on strong chemical interactions between a sensor and an analyte [21]. This approach is advantageous for discrimination of complex mixtures and overcomes some of the limitations associated with sensors that rely on non-specific interactions (*e*.*g*. physisorption) and depend on the physical traits of the analyte or on the surface features of the sensor [22].

The use of optical detection in combination with CSAs is particularly advantageous for indoor air pollution monitoring, due to its high sensitivity and the associated convenience of the instrumentation. A colour change can be detected visually in response to analyte interactions with certain reagents for a quick qualitative analysis, or reflectance detection used for quantitative analysis of these colour changes, whereby a desktop scanner or digital camera is used to capture the light reflected off the surface of the coloured sensors printed on an array. The red, green and blue (RGB) values of the spots are then determined using standard digital image analysis software. This detection method has the advantage of employing widely available technologies (*e*.*g*. digital camera or mobile phone camera) for ease of measurement outside the laboratory setting [15]. Lower-cost and easy-to-use air pollution sensors also provide opportunities for citizen science initiatives, which involves many individuals voluntarily collecting data that is later aggregated and analysed. This could provide citizens with opportunities to monitor their local air quality and to become more informed about air quality in their home and community.

We recently reported a colorimetric sensing approach to measure cooking associated air pollution indoors. This method was validated by gas chromatography-mass spectrometry (GC-MS) and it enabled discrimination of the complex mixtures of VOCs generated from cooking [7]. The present study aimed to bring this method from the laboratory into the field to enable measurement of VOCs in indoor air in households. The sensor array was designed to target VOCs commonly found in indoor air (carbonyls and organic acids) by incorporating pararosaniline, N,N-dimethyl-4,4’-azodianiline and pH indicators as sensing materials. Sensors were applied to the measurement of VOCs in the domestic setting to characterise household air pollution to investigate the response of these sensors to household locations and activity levels. A number of different rooms in a single household were monitored using the sensors over 24 h to understand the dynamic sensor response as a function of specific activities in the household. A broader sensor study across 9 different households in kitchen areas was also carried out and the data analysed to investigate links between sensor response, occupant activity and year of house build. This work serves to show the efficacy of the use of colorimetric sensor arrays for indoor air pollution monitoring in the domestic setting.

## Materials and methods

### Materials

Bromphenol blue, bromocresol green, thymol blue, methyl red, nitrazine yellow, methyltriethoxysilane, triethoxy(octyl)silane, 2-methoxyethanol, propylene glycol methyl ether acetate, polyethylene glycol *tert*-octylphenyl ether, pararosaniline base, N,N-dimethyl-4,4’-azodianiline, *p*-toluenesulfonic acid and sulfuric acid were purchased from Sigma-Aldrich (Arklow, Co. Wicklow, Ireland). Analytical standards were also purchased from Sigma-Aldrich, Ireland. Substrates (Polygram® CEL 300) were obtained from Machery-Nagel GmbH (Düren, Germany). Water used was high purity Milli-Q water (Millipore >18 MΩcm). A vacuum sealer (iLmyh, model number ZS-11W2) and vacuum sealer bags (Culivac, B16025S) were used for sensor storage and shipment.

### Preparation of sensors

Pararosaniline and *N*,*N*-dimethyl-4,4’-azodianiline dyes were mixed with one of two acids (sulfuric or p-toluenesulfonic acid) in different molar ratios and dissolved in a plasticizer (polyethylene glycol *tert*-octylphenyl ether). 4 mg of each pH indicator was dissolved in 1 mL of a sol gel. The sol gel was prepared by mixing silane pre-cursors (methyltriethoxysilane and triethoxy(octyl)silane) with 2-methoxyethanol, propylene glycol methyl ether acetate, deionised water and a catalyst (0.1 M hydrochloric acid) in a molar ratio of 1:1:25:10:70:0.05. This mixture was stirred overnight at room temperature to yield a sol gel. The dyes and formulations used in each sensor spot are shown in S1 Fig. During initial development and laboratory testing of the sensor array, the 16 formulations were transferred to 16 cm^2^ substrates using a micropipette. For subsequent experiments sensor formulations were deposited into a 96-well plate and sensor arrays were prepared by using a microplate replicator (Boekel Scientific, PA, USA) to transfer reactive spots to 16 cm^2^ substrates, constructing a 4 x 4 sensor array. Sensor arrays were dried under vacuum overnight and then stored under vacuum until use.

### Analysis of images

A digital scanner was used to capture images of sensor arrays before and after exposure to standard analytes and indoor air. Red (R), green (G) and blue (B) values were measured for each sensor spot before and after exposure using Digital Colour Meter software (Version 5.13, Apple Inc.). Averaged RGB values were recorded from a 5 x 5 pixel area on each sensor spot. The colour change (ΔRGB) was calculated by taking the difference of the R, G and B values before and after exposure to an analyte or sample. The response for each sensor was calculated using the Euclidean distance (ΔE) formula shown in Eq 1, where ΔR, ΔG and ΔB are the differences between the red, green and blue components, respectively, in the images collected before and after exposure.


$$Euclidean\;distance=\sqrt{\left(\Delta R^{2}+\Delta G^{2}+\Delta B^{2}\right)}$$ (1)

Microsoft Excel and RStudio were used for data processing and analysis. R packages factoextra, FactoMineR and ggplot2 were used for data analysis and generation of figures.

### Characterisation of colorimetric sensor response to standard vapours

Standard analytes and colorimetric sensors were loaded into 1.8 L glass chambers for measurement of sensor response. Liquid analytes were dropped onto filter paper which was suspended at the top of the test chamber. The sensor arrays were placed at the bottom of the chamber to detect analyte vapours. The chamber was closed, and sensor readings were recorded after a 2 h period inside the chamber. A digital scanner was used to capture images of the sensor array at the start and end of the exposure period. Control measurements were collected in the absence of any analytes in the test chambers, and experiments were repeated in triplicate. The sensor array was applied to a quantitative analysis of different concentrations of a single aldehyde (pentanal) and acid (acetic acid). Responses to acetic acid were calculated as the sum of ΔE values for sensors 1–6 (shown in S1 Fig) and responses to pentanal were calculated as the sum of ΔE values for sensors 7–16 (shown in S1 Fig).

### Monitoring the impact of occupant activities on household air pollution using colorimetric sensor arrays

Colorimetric sensor arrays were vacuum sealed for storage and transport to a single household for testing of sensor performance. Sensors were deployed in an open plan living room/kitchen for a 1 week period. Images of the sensors were taken using a digital scanner at the start of the study (0 h) and at various time points over the course of the week (15, 24, 30, 40, 48, 60, 72, 84, 115 and 168 h). Control sensors were stored under vacuum in the same room for the duration of the study and were imaged at the same time points.

The potential influence of occupant activity on sensor response during periods of occupancy was further investigated during a 48 h period. Sensors were deployed in an open plan living room/kitchen, a home office, a bedroom and its en suite bathroom to compare responses obtained for the different room environments. Images of the sensors were collected at the start and end of the 48 h study period and sensor responses were calculated using Eq 1.

Sensors were also deployed in an open plan living room/kitchen and an office for stationary sampling over a 48 h period. Sensors were imaged once every hour over a 12 h period during the day and a log book detailing occupant activities was kept during the study period. This study focused on an open plan kitchen/living room as a potentially high VOC environment and a home office as a potentially low VOC environment. The change in sensor response with respect to time was calculated using Eq 2.

$$First\;derivative\;of\;sensor\;response=\frac{y_{2}-y_{1}}{x_{2}-x_{1}}$$ (2)

where x is time elapsed (h) and y is sensor response (ΔE)

Two parallel sensor samples were subsequently collected from 9 domestic kitchens over a 48 h sampling period. Sensor images were scanned at time points 0 and 48 h. The year the house was built was recorded, as well as the occurrence of the activities that could potentially contribute to airborne VOCs during the 48 h sampling period, including smoking, cleaning, use of personal care products and cooking. Principal component analysis (PCA) was performed on the full dataset generated in this study including quantitative variables (sensor responses, year built) and qualitative variables (cleaning, smoking, personal care products). Qualitative variables were assigned a value of 1 or 0 for the purpose of performing PCA on the dataset. A value of 1 was assigned where an activity occurred during the sampling period. A value of 0 was assigned where an activity did not occur during the sampling period.

## Results and discussion

### Initial characterisation of sensor performance

#### Characterising sensor response to standard vapours

**Fig 1:**
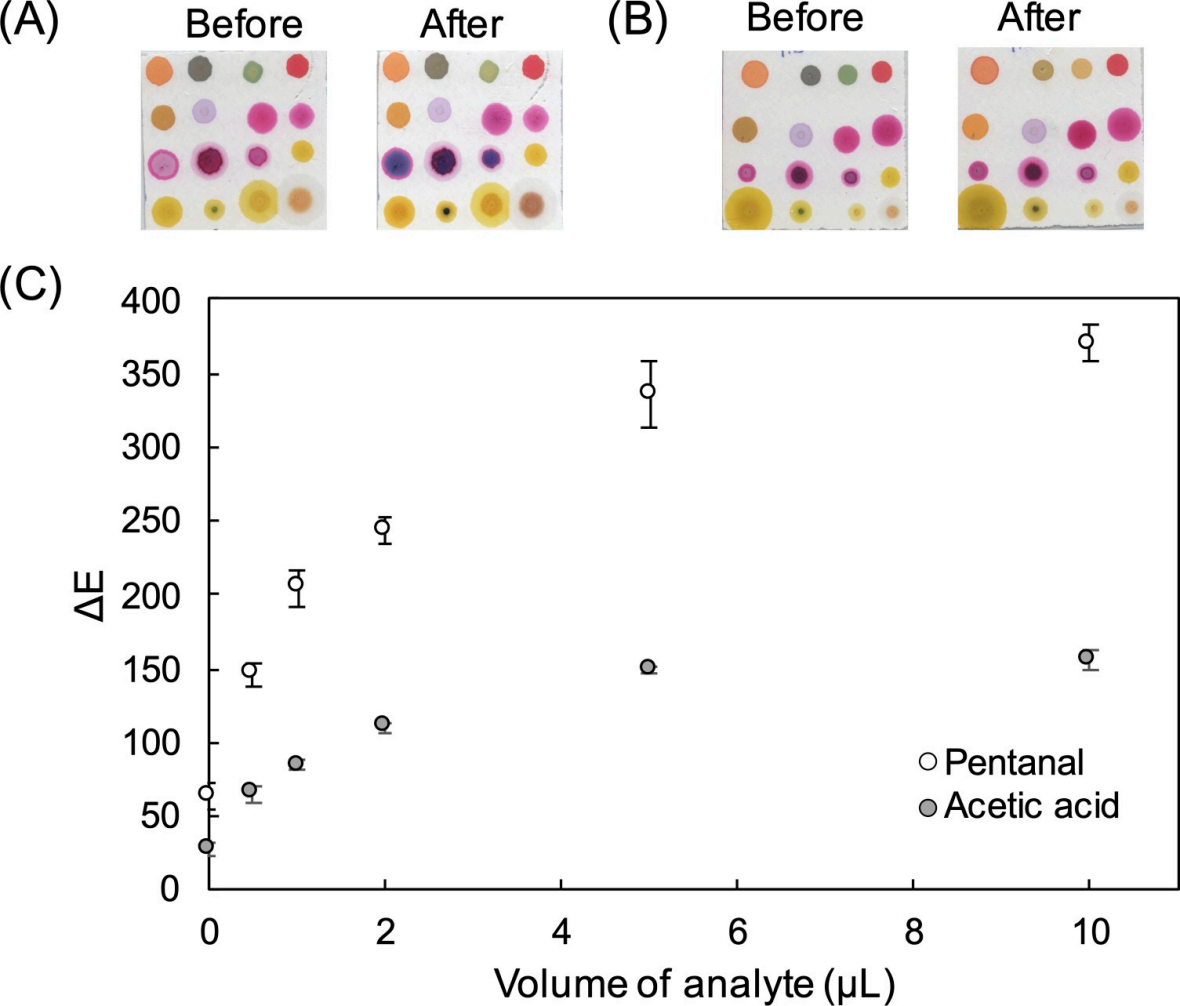
Scanned images of sensor array taken before and after exposure to (A) pentanal vapour and (B) acetic acid vapour; (C) graph shows ΔE (Euclidean distance) for carbonyl sensors to increasing amounts of pentanal and pH sensors to increasing amounts of acetic acid. Error bars represent the standard deviation of triplicates.

The colorimetric sensor array is designed to show a selective response to acids, aldehydes, and ketones and its overall responses to indoor air cooking emissions have been validated earlier by GC-MS analysis [7]. The array incorporates pH indicators and amine-containing indicators targeting nucleophilic addition to a carbonyl group by an amine in the formation of an imine. The sensor array contains 16 individual sensor spots, as shown in Fig 1(A), where the amine-containing indicators underwent a visually detectable colour change after exposure to pentanal vapour. Specifically, the pararosaniline sensors changed from a pink to a purple colour, and the azodianiline sensors changed from a yellow to an orange colour. The pH indicators underwent a visually detectable colour change after exposure to acetic acid vapour (Fig 1(B)). Digital imaging of the sensor array before and after exposure to standard vapours enabled quantitative measurement of red, green and blue colour values for each sensor spot. The response of the sensor array to increasing amounts of the 2 standard vapours was also investigated. Sensor response (ΔE) was calculated using Eq 1, and this value was plotted against the volume of analyte loaded into the test chamber. The resultant calibration curves are shown in Fig 1(C) where ΔE increases gradually with the increasing concentrations of each analyte. The colour change of the sensor array responded proportionally to the amount of valeraldehyde and acetic acid up to approximately 5 μL before reaching saturation, at which point there is a plateau in the graph. The carbonyl sensors (7–16) showed a wide response range to pentanal (ΔE up to approximately 350) while sensors 1–6 had a more moderate range (ΔE up to approximately 150) in response to acetic acid. The colour change of the sensor array responded proportionally to the concentration of pentanal and acetic acid meaning that the CSA could be employed for analysis of aldehydes and acids over a broad range of concentrations, and even for semi-quantitative analysis using the corresponding fitting curves between ΔE and analyte concentration.

### Sensor deployment in one household

#### Investigating sensor performance in the household environment

Colorimetric sensor arrays were vacuum sealed for storage and transport to a single household for testing of sensor performance. Sensors were deployed in an open plan living room/kitchen for a 1 week period. Images of the sensors were taken at the start of the study (0 h) and at various time points over the course of the week. Control sensors were stored under vacuum in the same room for the duration of the study and were imaged at the same time points. Sensor response (ΔE) was calculated using Eq 1, and sensor responses for the pH indicators and amine containing indicators with respect to time are shown in S2A and S2B Fig respectively. The control sensors showed a slight increase in ΔE values between 0 and 60 h due to aging of the sensor materials. Both the pH indicators and amine containing indicators underwent colour changes upon exposure to indoor air. There was a steep increase in ΔE values observed during the first 48 h of the study period. After 48 h, the acid sensors reached a saturation point, as evidenced by the plateau in S2A Fig. After 48 h, the aldehyde sensors continued to show increasing responses, although this was more gradual than that observed during the initial 48 h (S2B Fig). Based on these results, 48 h was selected as a suitable time period for subsequent sensor deployments in home settings.

Sensors were deployed in different rooms within the same household to investigate the capability of the sensor array to measure differences between rooms. Sensors were deployed in an open plan living room/kitchen, a home office, a bedroom and its en suite bathroom. Within the open plan living room/kitchen, sensors were placed in the cooking area of the kitchen, and at the opposite end of the room in the living room area. Images of the sensors were taken at the start and end of the 48 h study period and sensor responses were calculated. S3A and S3B Fig show the total response for the pH and amine containing indicators respectively. Both groups of sensors underwent a colour change after exposure to air in all 5 rooms suggesting the presence of volatile acids and carbonyl compounds. The pH indicators underwent the greatest colour change in the bathroom and bedroom, suggesting that there were higher levels of acids compared to the other rooms. This may be linked to occupancy and activities (*e*.*g*. use of personal care products). The pH indicators also underwent colour changes in the living room and kitchen, where occupancy and cooking activities may have contributed. Organic acids have several indoor sources including building materials, household products, human metabolism and oxidation chemistry [23]. Sorbic acid is used as an ingredient in personal care products, and may have contributed to the high sensor responses observed for the bathroom where various personal care products were in use. Acetic acid and formic acid are associated with human occupancy in addition to off-gassing from building materials and household products. Lactic acid and sorbic acid have been linked to occupancy levels and cooking activities, while benzoic acid is associated with cooking [8]. The amine containing indicators underwent the greatest colour change in the kitchen (S3B Fig) which may be linked to the emission of carbonyl compounds during cooking activities [24]. High responses were also observed for sensors deployed in the living room, bedroom and bathroom where occupant activities such as cooking, cleaning and the use of consumer products, cleaning, *etc*. may have contributed to higher levels of carbonyl compounds compared to the office. The lowest sensor responses were observed in the office, where the only activity during periods of occupancy was computer-based work.

**Fig 2:**
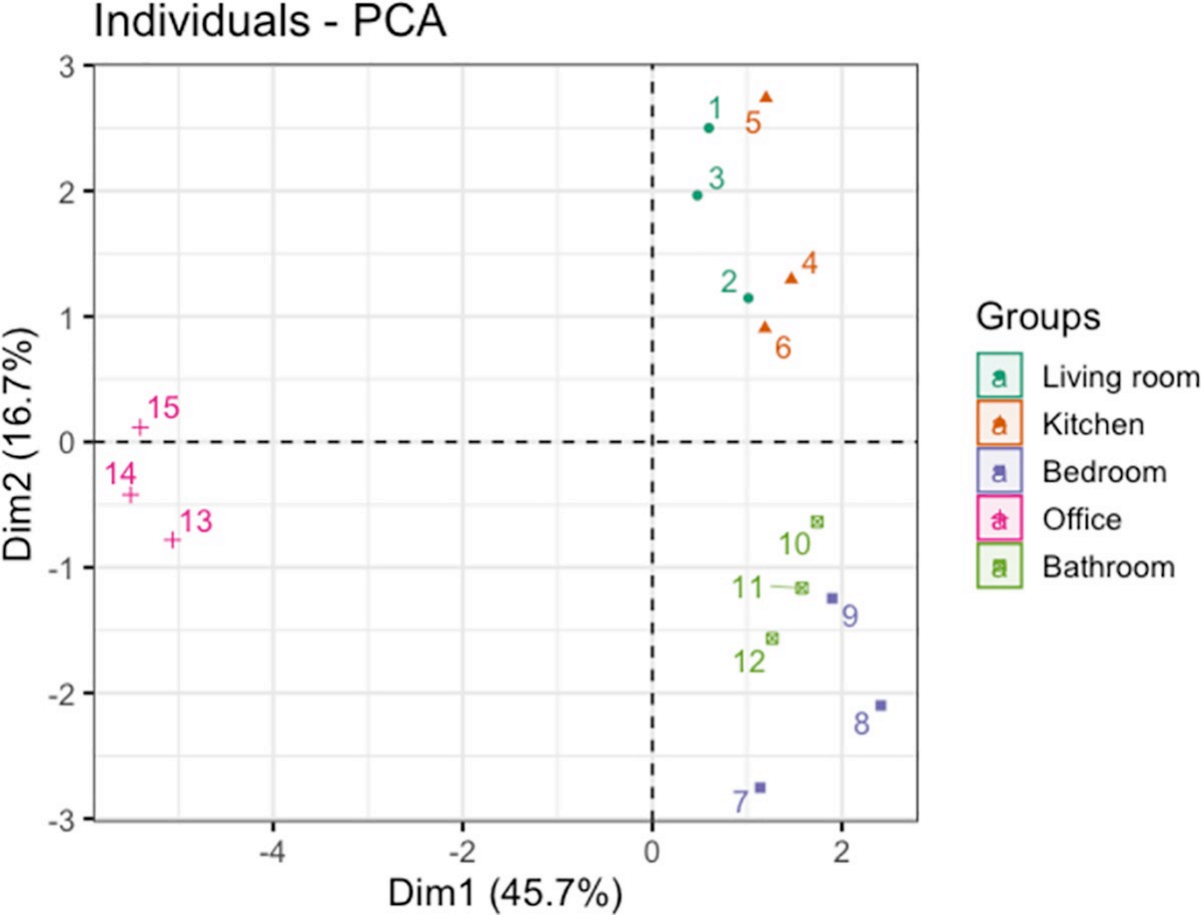
Scores plot from PCA on sensor array responses after 48 h deployment in different rooms within a single home.

Principal component analysis (PCA) was performed on the individual ΔE values for each sensor spot on the array (240 ΔE values) to understand the capability of the sensor array to measure differences between rooms. Fig 2 shows the scores plot from PCA, where the rooms were classified into 3 groups. The office was separated from the other rooms with a negative score on principal component 1 (PC1). The living room, kitchen, bedroom and bathroom all showed positive scores on PC1. This separation may be due to the overall difference in sensor responses (low in office, high in other rooms). S4 Fig shows that several of the pH (sensors 2–4) and amine-containing indicators (sensors 7–9, 11, 13–15) made important contributions to the ability of the sensor array to discriminate between the office and the other rooms, and a higher score on PC1 indicates a stronger interaction between a sample (*i*.*e*. the air in a room) and these sensors, as measured by the intensity of the sensor response. The living room and kitchen were separated from the bedroom and bathroom on PC2 based on their different interactions with both pH indicators and amine-containing indicators on the sensor array. S5 Fig shows that sensors 5, 10, 12, 13 and 16 made an important contribution to the ability of the sensor array to discriminate between the living room/kitchen and the bedroom/en suite bathroom. A low score on PC2 corresponds to a higher response for sensor 5 (which was observed for the bedroom and bathroom) whereas a higher score on PC2 indicates a stronger interaction between a sample and sensors 10, 12, 13 and 16 (as observed for the kitchen and living room).

#### Investigating the influence of activity levels on household air pollution and on sensor response

**Fig 3:**
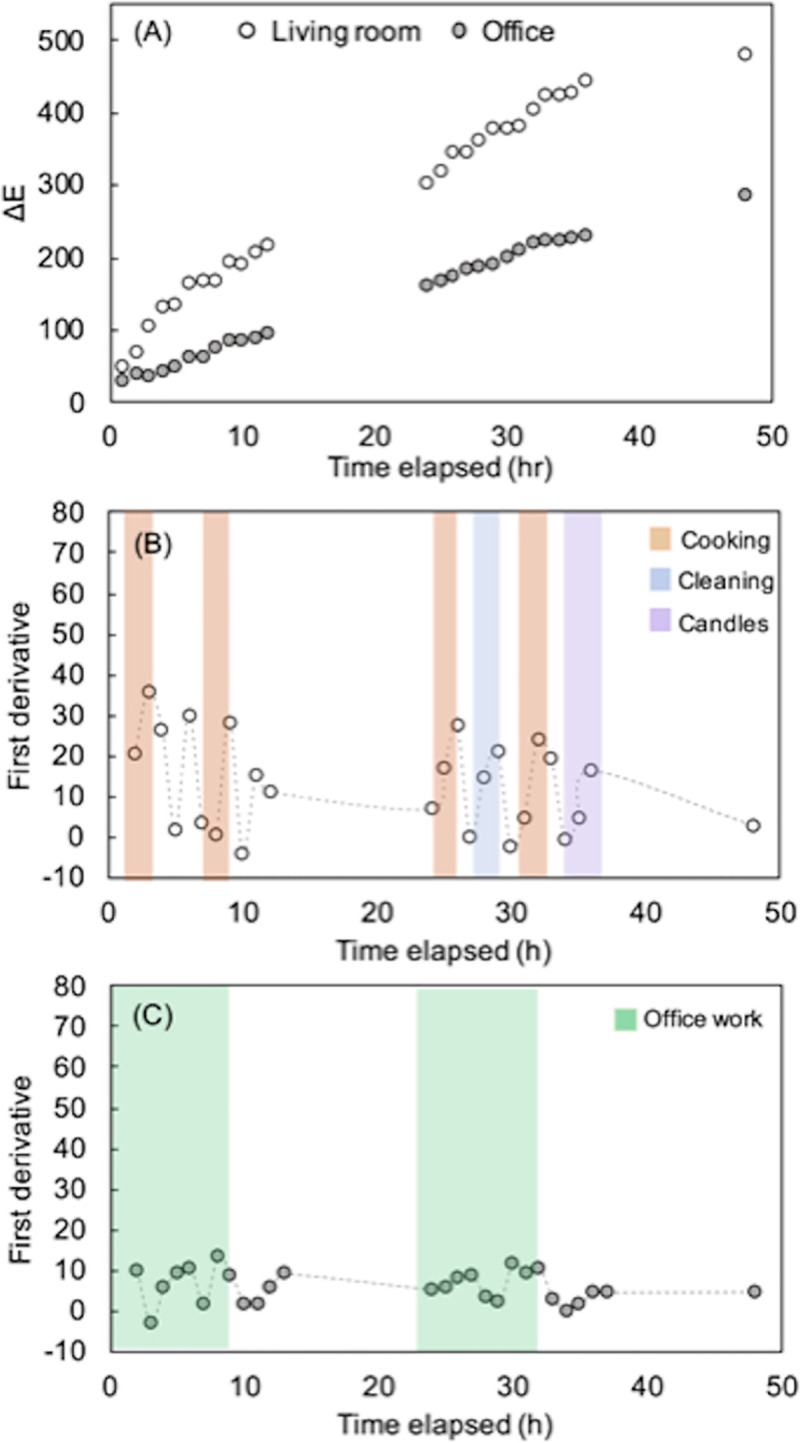
(A) Cumulative response of sensor array during 48 h deployment in open-plan living room/kitchen and office; (B), (C) first derivative of sensor response shows the impact of different types of activities on VOC levels indoors.

The potential influence of occupant activity on sensor response during periods of occupancy was further investigated. Sensors were placed in an office and in an open plan kitchen/living room for 48 h. These two rooms were selected for further investigation as they were the main rooms that were occupied in the household during the daytime. The kitchen represented a high-pollution room where regular cooking and cleaning activities took place, and the office represented a low-pollution room where the only activity was computer-based work. More frequent imaging of sensors was employed during the 48 h study in order to understand the influence of occupant activity on sensor responses. Sensor imaging took place once every hour during the daytime while the rooms were occupied (between 9 am and 10 pm) and a logbook of occupant activities was kept during the study. Sensor response (ΔE) was calculated using Eq 1, and sensor responses with respect to time are shown in Fig 3(A). The sensors underwent a colour change in both rooms, suggesting the presence of volatile acids and carbonyl compounds in the air. Fig 3(A) shows a steep increase in ΔE for the kitchen/living room, whereas a more gradual increase in sensor response was observed for the office. The change in sensor response with respect to time can be obtained by calculating the first derivative of sensor response (Eq 2). The first derivative of sensor response for the kitchen/living room and office are shown in Fig 3(B) and 3(C) respectively. The periods of occupant activity recorded in the log book are overlaid in Fig 3(B) and 3(C), where each activity is represented by a different colour. The smallest changes in sensor responses for any activity was observed in the office, where office work related to single person occupancy computer-based work. Despite the colour changes measured being small, the activity was still identifiable on account of it resulting in a change in the VOC composition in the room.

Colorimetric sensor arrays present an advantage over conventional approaches to VOC monitoring in households as they enable collection of temporal VOC data on specific classes of compounds. Traditional approaches to collect household VOC data rely on sampling at a single point in time. This can provide high quality data but limits the collection of temporal data that’s needed to characterise occupant impacts on indoor air pollution. Improving knowledge of the human factors influencing air pollution in addition to the temporal changes that occur on a day-to-day basis would allow for better understanding of human exposure and improvement of remediation strategies.

### Field study—sensor deployment in different households to characterise ambient VOC air pollution

A pilot field study was conducted using colorimetric sensor arrays to monitor ambient VOCs in different households. The field study focused on residential kitchens where cooking is a prominent source of VOCs, including volatile acids, and carbonyl compounds, such as aldehydes. These pollutants are of particular concern to human health as irritants of the eyes, skin and respiratory tract and their potential carcinogenicity [25, 26]. Air pollution generated during cooking can persist in the home for several hours [27], making this an issue of concern to people who routinely cook in domestic settings, and particularly where ventilation systems are lacking or under-utilised.

Sensors were deployed in 9 different households in Kuopio, Finland. Two sensors ((a) and (b)) were placed in the kitchen area of each household for a 48 h study period. All sensors were deployed over the same 48 h period in February 2020. Sensors were imaged at 0 h and 48 h using a digital scanner. Control sensors were stored under vacuum for the duration of the study and were imaged at the same time points. Sensor response (ΔE) for each spot was calculated using Eq 1. Occupant activities were also logged during the study period and the year each house was built was recorded as supporting data. Positive sensor responses were observed for all households, with both acid and carbonyl sensors showing a response after the 48 h deployment.

**Fig 4:**
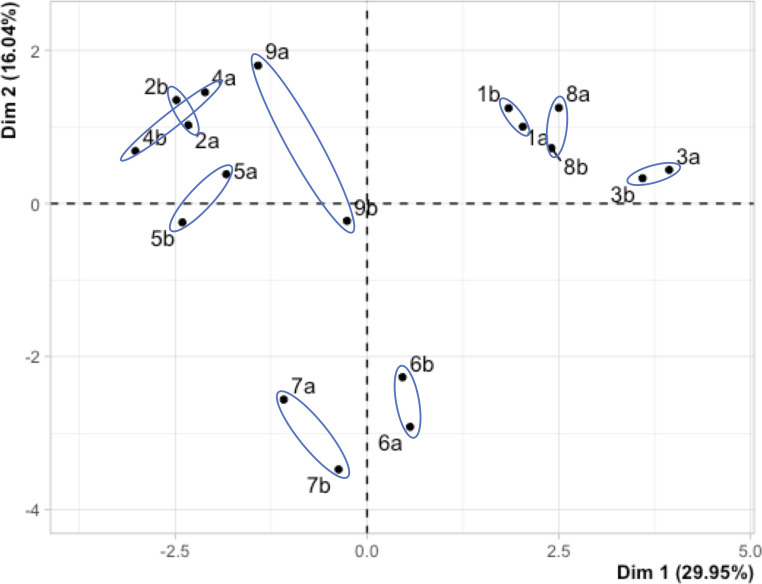
Scores plot from PCA on sensor array responses after 48 h deployment in 9 households. Two sensors were deployed in each household (labelled (a) and (b)) and are linked by the blue ovals overlaid on the plot.

PCA was performed on the individual ΔE values for each sensor spot on the array (144 ΔE values). Fig 4 shows the scores from PCA, where the samples were classified into 3 groups. Duplicate samples collected from each household (sensor (a) and sensor (b)) were clustered together owing to the similar colorimetric responses obtained for sensors deployed in the same location.

PC1 distinguished between samples primarily based on the strength of their interaction with acid and carbonyl sensors. A positive score on PC1 was associated with a higher carbonyl sensor response, as seen for samples 1, 3, 6 and 8. A negative score on PC1 was linked to higher acid sensor response, as observed for samples 2, 4, 5, 7 and 9. Samples 6 and 7 were separated from all other samples with negative scores on PC2. This separation may be due to the overall difference in sensor responses; the lowest responses were observed for samples 6 and 7. The contribution of all variables (sensors) to PC1 and PC2 is shown in a biplot (S9 Fig) which highlights the important roles played by the majority of sensors tested in distinguishing between the acids and carbonyl-based compounds detected. The individual carbonyl sensor responded predominantly in the positive PC1 direction while the individual acid sensor responses were all in the negative PC1 direction.

**Fig 5:**
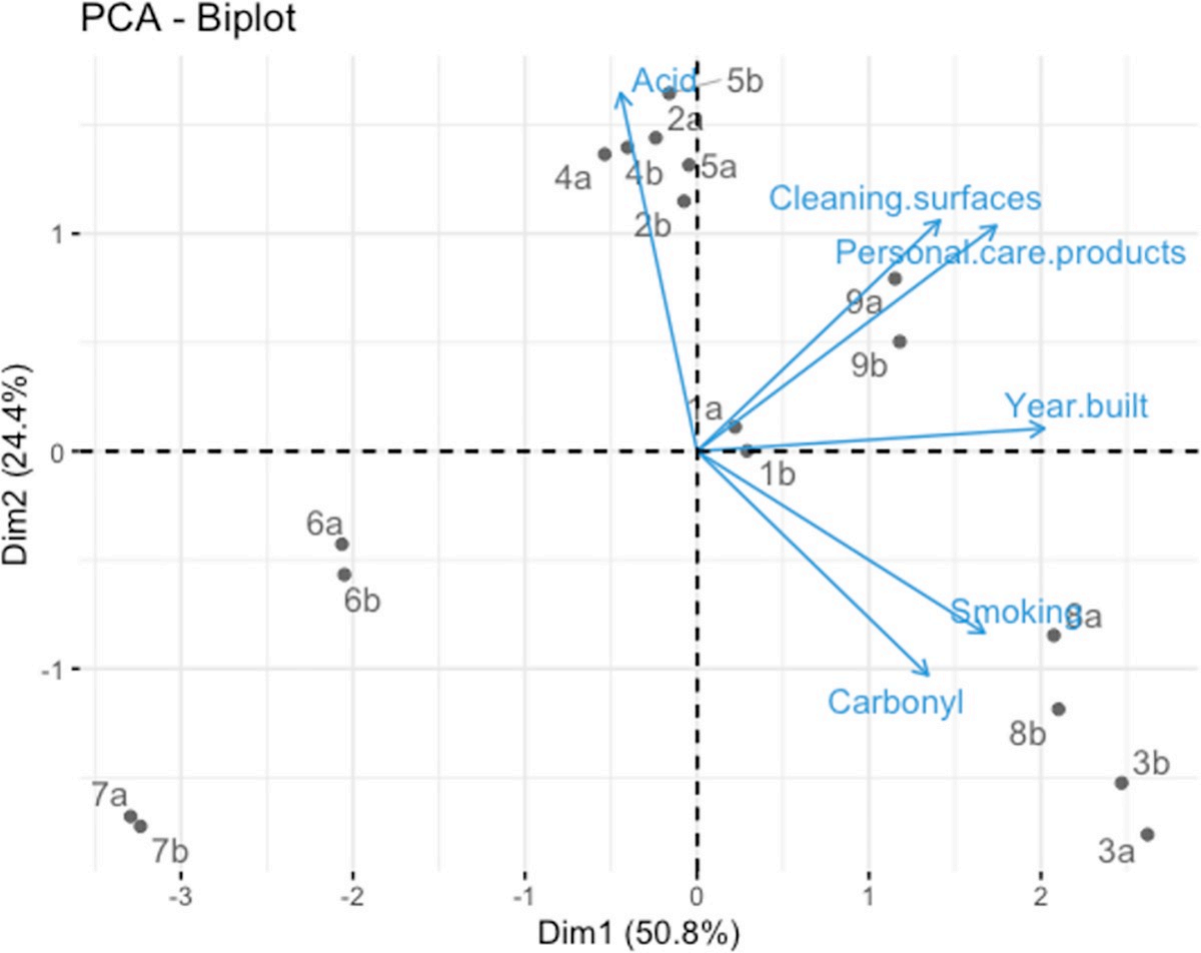
Biplot from PCA on sensor array responses after 48 h deployment in 9 households. The arrows show the variables (acid sensors, carbonyl sensors, cleaning, use of personal care products, smoking, year built). The length of the arrows represent the contribution of each variable to PC 1 and 2. Two sensors were deployed in each household (labelled (a) and (b)).

PCA was also performed on the full dataset collected in this study, incorporating occupant activity and year built data (S2 Table) and sensor response (S3 Table), as variables in order to investigate the nature of the clustering of the data with all variables considered. The sum of sensor responses for acids (sensors 1–6) and aldehydes (sensors 7–16) were used in combination with the activity and year built data in order to provide an overview of the data. Fig 5 shows the biplot from PCA, where the samples were classified into several different groups. Separation on PC1 was strongly linked to the year each house was built. The 3 most modern houses (3, 8 and 9—all built between the years 2008 and 2016) showed the highest scores on PC1. The lowest scores on PC1 were observed for the oldest buildings (namely 6 and 7, which were built in the years 1922 and 1928, respectively). More modern builds were associated with higher scores on PC1, which may potentially be linked to the ventilation system or the amount of new materials in the house, however, these parameters were not assessed within the scope of this study [28]. Separation on PC2 was partially linked to sensor responses to different compound classes. The highest scores on PC2 were observed for the houses where the greatest sensor response to volatile acids was observed (houses 2, 4, and 5). Higher responses for carbonyl sensors were observed for houses 3 and 8, which both had a negative score on PC2. These homes also reported smoking in the kitchen, an activity that is known to generate numerous volatile carbonyl compounds [29]. House 9 separated from other houses on PC2 whereby the use of cleaning and personal care products influenced this separation. This house showed high sensor responses for both acid and carbonyl compounds (S3 Table), likely linked to these activities as well as to smoking. Note that cooking as a variable was not considered in this analysis as all houses reported cooking activity over the 48 h sampling period and therefore could not be used as a qualitative variable to discriminate between samples.

Overall, we can see that although PC1 is largely influenced by year of build, the houses remain clustered in a similar manner as they did for Fig 4 where only the sensor responses were used as variables. This further confirms the strong correlation between year of build and colorimetric sensor response. It can also be seen in the data that the different occupant activities during the time of sensor deployment influenced the sensor response whereby smoking resulted in high carbonyl sensor responses while the use of personal care products and cleaning resulted in greater acid responses.

## Conclusion

There is a huge need for easy to use, fast and economical detection systems to monitor the various types and amounts of air emissions from different sources in the home. The sensor array described here, comprising pH indicator and aniline dyes, can enable molecular recognition of a variety of carboxylic acids and carbonyl compounds, which were shown in this work to be responsive to emission sources arising from day-to-day household activities including cooking and cleaning. The temporal nature of the data that can be collected using this approach could enable it as a viable solution to real-time monitoring of indoor air quality in the home as part of exposure monitoring and human health risk assessment. Furthermore, when sensor arrays were deployed in a number of households, the sensor response was shown to correlate with factors such as occupant activity and year of house build (potentially relating to ventilation efficiency or building materials), highlighting the potential for these easy-to-use sensor arrays to provide real-time data to the occupants to inform on temporal ventilation needs. In terms of limitations to the present study, certain results may have been influenced by variability in environmental conditions between households (e.g. changes in ambient temperature or humidity during sampling) or sensor saturation in high exposure situations. Future work will focus on greater numbers of deployments of these sensors for collection of larger datasets across different household settings (e.g. urban vs rural, occupant compositions, etc) with greater temporal resolution, to further demonstrate the facile deployment of such sensors and their potential for tracking indoor air quality in the home in a simple and visual manner.

## Supporting information

S1 Fig Image of colorimetric sensor array and list of materials used in each sensor spot. (PDF)

S2 Fig Sensor response (ΔE, Euclidean distance) over a 1 week period for pH sensors (A) and carbonyl sensors (B). Sensors were deployed in an open-plan living room/kitchen (open marker) and the control sensors (shaded marker) were stored under vacuum in the same room for the duration of the study.
(PDF)

S3 Fig Sensor response (ΔE) to volatile acids (A), aldehydes and ketones (B) during 48-hour deployment in different rooms within a single home (error bars represent standard deviation of triplicates).
(PDF)

S4 Fig Bar chart showing the contributions (%) of individual sensors on the array to principal component 1. The red dashed line indicates the expected average contribution of a sensor. For a given component, a sensor with a contribution larger than this cut-off can be considered as important in contributing to the component.
(PDF)

S5 Fig Bar chart showing the contributions (%) of individual sensors on the array to principal component 2. (PDF)

S6 Fig Sensor response (ΔE, Euclidean distance) over a 48 h period for pH sensors (A) and carbonyl sensors (B) deployed in open-plan living room/kitchen and office.
(PDF)

S7 Fig First derivative of sensor response over a 48 h period for pH sensors (A) and carbonyl sensors (B) deployed in an open plan living room/kitchen.
(PDF)

S8 Fig First derivative of sensor response over a 48 h period for pH sensors (A) and carbonyl sensors (B) deployed in an office.
(PDF)

S9 Fig Biplot from principal component analysis on sensor array responses after 48 h deployment in 9 households. The arrows show the variables (sensors 1 to 16). The length of the arrows represent the contribution of each sensor to principal components 1 and 2. Two sensors were deployed in each household (labelled (a) and (b)).
(PDF)

S10 Fig Biplot from principal component analysis on full data set after 48 h deployment in 9 households. The arrows show the variables (sensors 1 to 16 and supporting information on cleaning, use of personal care products, smoking, year built). The length of the arrows represent the contribution of each sensor to principal components 1 and 2. Two sensors were deployed in each household (labelled (a) and (b)).
(PDF)

S1 Table Inter-sensor variability shown for triplicate sensor arrays deployed in open plan living room-kitchen for 72 h. (PDF)

S2 Table Supporting information collected during sensor deployment in 9 households. (PDF)

S3 Table Average sensor responses obtained after 48 h deployment in 9 different households. (PDF)
