# Supplementary material for: Visualising household air pollution: Colorimetric sensor arrays for monitoring volatile organic compounds indoors
Source: PLoS One. 2021 Oct 6;16(10):e0258281. doi: 10.1371/journal.pone.0258281 (PMC8494322; doi:10.1371/journal.pone.0258281)
Supplement: S1 Fig — (PDF) [file pone.0258281.s001.pdf]

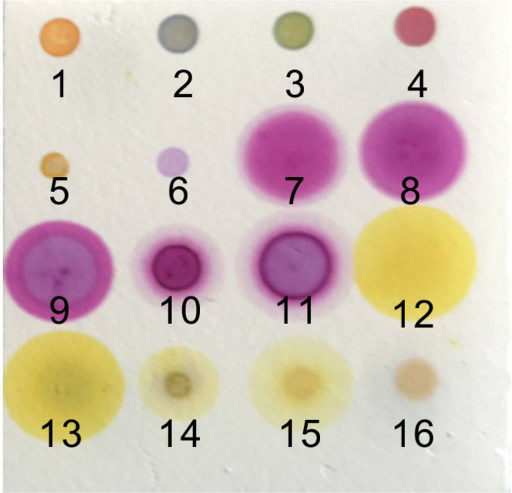

1	Thymol blue
2	Bromophenol blue
3	Bromocresol green
4	Methyl red
5	Nitrazine yellow
6	Nile red
7	Pararosaniline
8	Pararosaniline:2 M TsOH (2:1)
9	Pararosaniline:2 M TsOH (1:2)
10	Pararosaniline:2 M H_2SO_4 (2:1)
11	Pararosaniline:2 M H_2SO_4 (1:2)
12	4,4'-Azodianiline
13	4,4'-Azodianiline:2 M TsOH (2:1)
14	4,4'-Azodianiline:2 M TsOH (1:2)
15	4,4'-Azodianiline:2 M H_2SO_4 (2:1)
16	4,4'-Azodianiline:2 M H_2SO_4 (1:2)
