# Supplementary figures and images for: Visualising household air pollution: Colorimetric sensor arrays for monitoring volatile organic compounds indoors
Source: PLoS One. 2021 Oct 6;16(10):e0258281. doi: 10.1371/journal.pone.0258281 (PMC8494322; doi:10.1371/journal.pone.0258281)

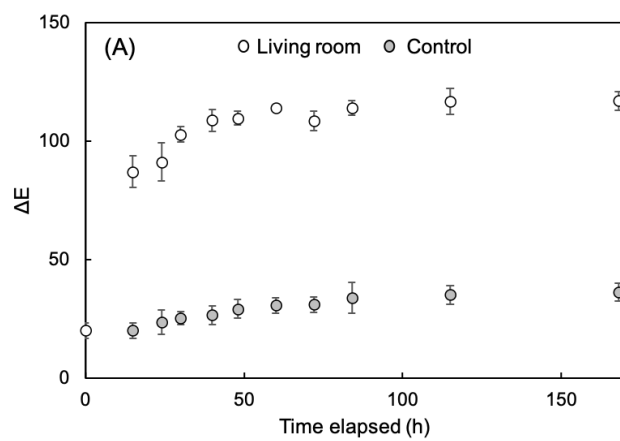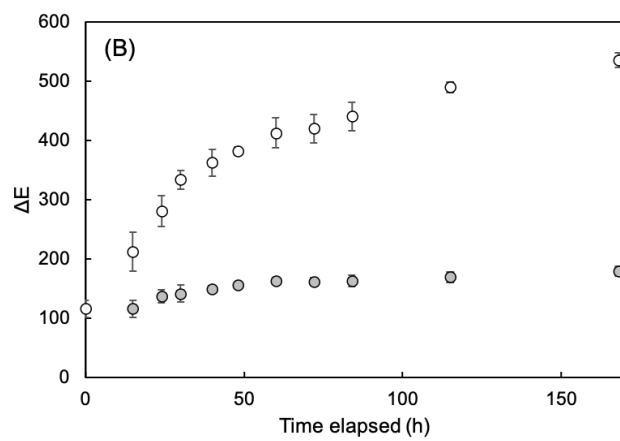

Supplement: S2 Fig — Sensor response (ΔE, Euclidean distance) over a 1 week period for pH sensors (A) and carbonyl sensors (B). Sensors were deployed in an open-plan living room/kitchen (open marker) and the control sensors (shaded marker) were stored under vacuum in the same room for the duration of the study. (PDF) [file pone.0258281.s002.pdf]

(A)

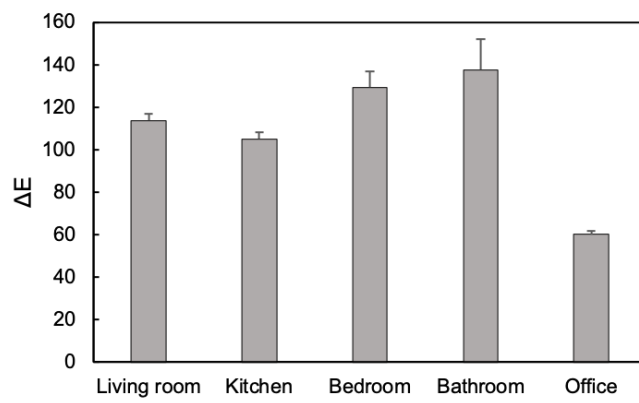

(B)

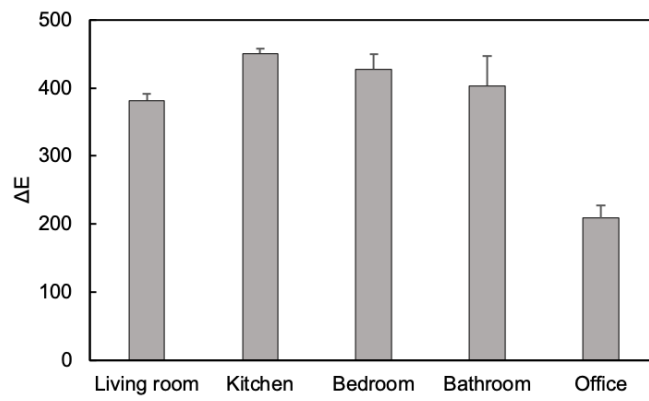

Supplement: S3 Fig — Sensor response (ΔE) to volatile acids (A), aldehydes and ketones (B) during 48-hour deployment in different rooms within a single home (error bars represent standard deviation of triplicates). (PDF) [file pone.0258281.s003.pdf]

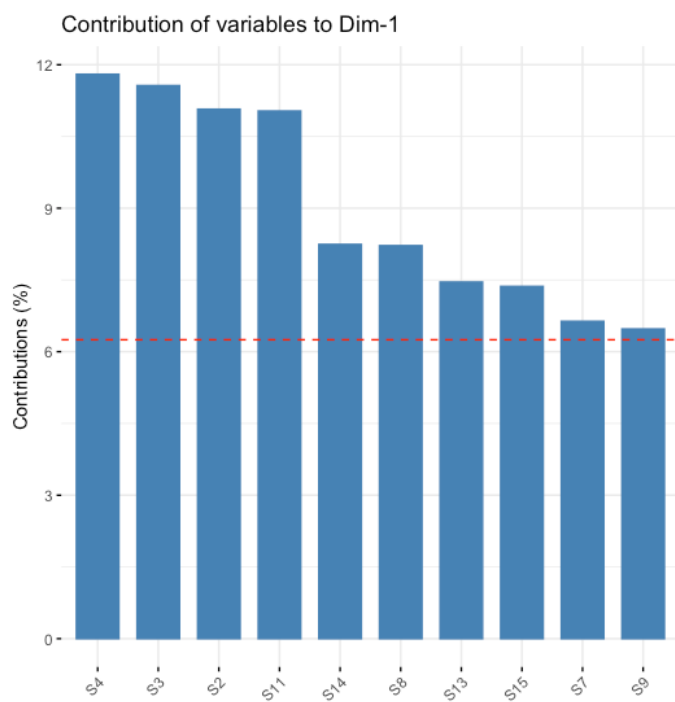

Supplement: S4 Fig — The red dashed line indicates the expected average contribution of a sensor. For a given component, a sensor with a contribution larger than this cut-off can be considered as important in contributing to the component. (PDF) [file pone.0258281.s004.pdf]

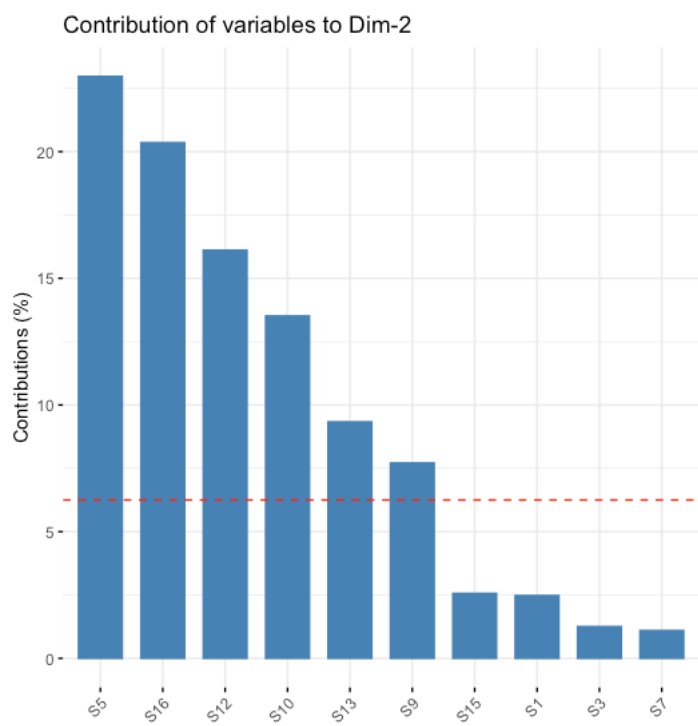

Supplement: S5 Fig — (PDF) [file pone.0258281.s005.pdf]

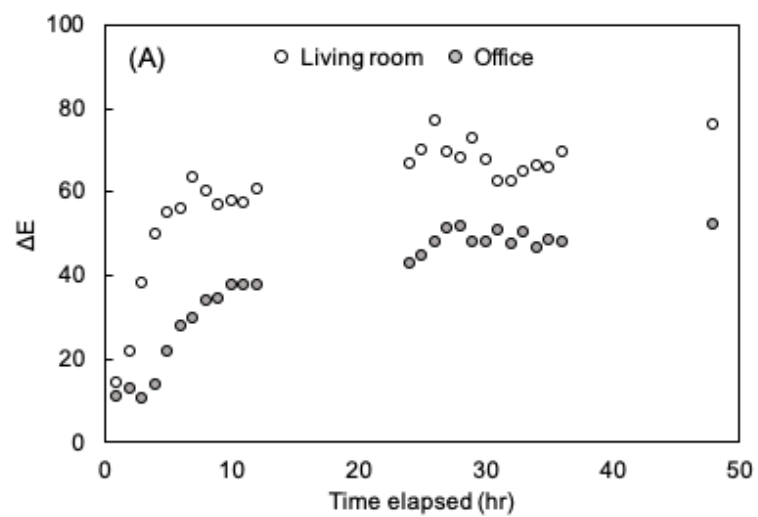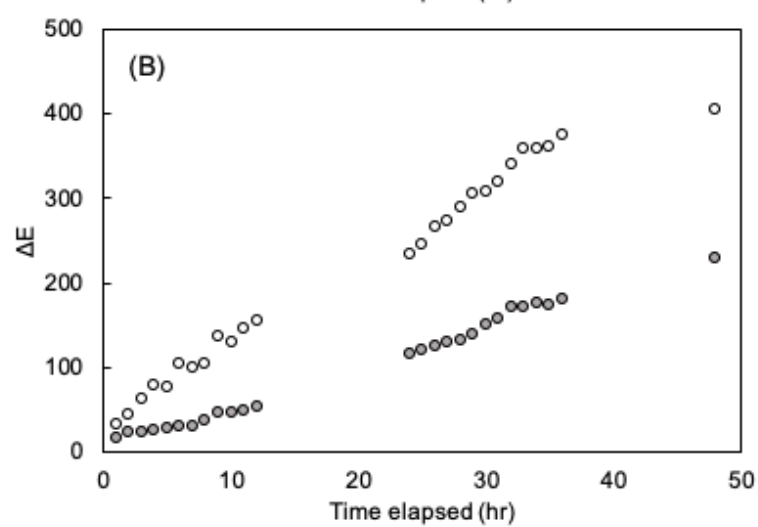

Supplement: S6 Fig — Sensor response (ΔE, Euclidean distance) over a 48 h period for pH sensors (A) and carbonyl sensors (B) deployed in open-plan living room/kitchen and office. (PDF) [file pone.0258281.s006.pdf]

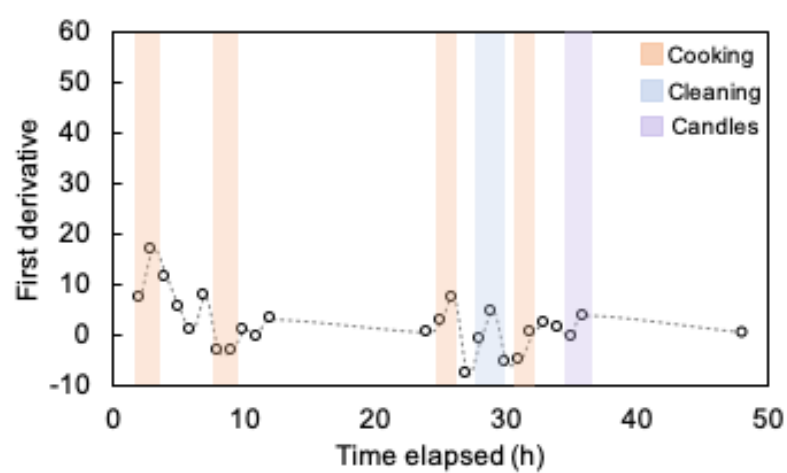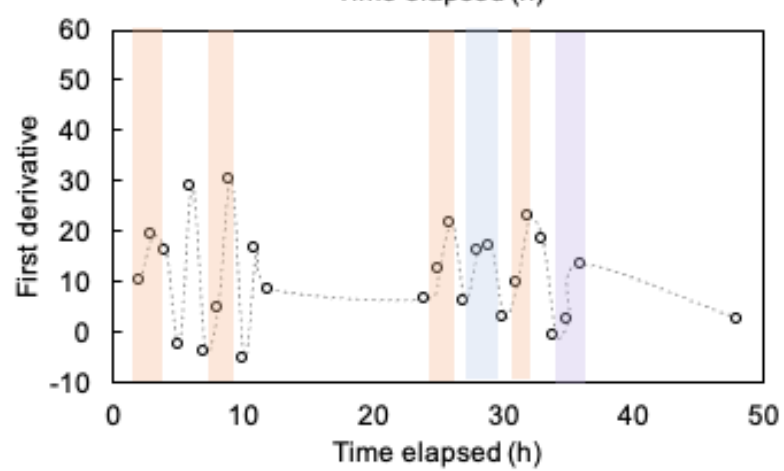

Supplement: S7 Fig — First derivative of sensor response over a 48 h period for pH sensors (A) and carbonyl sensors (B) deployed in an open plan living room/kitchen. (PDF) [file pone.0258281.s007.pdf]

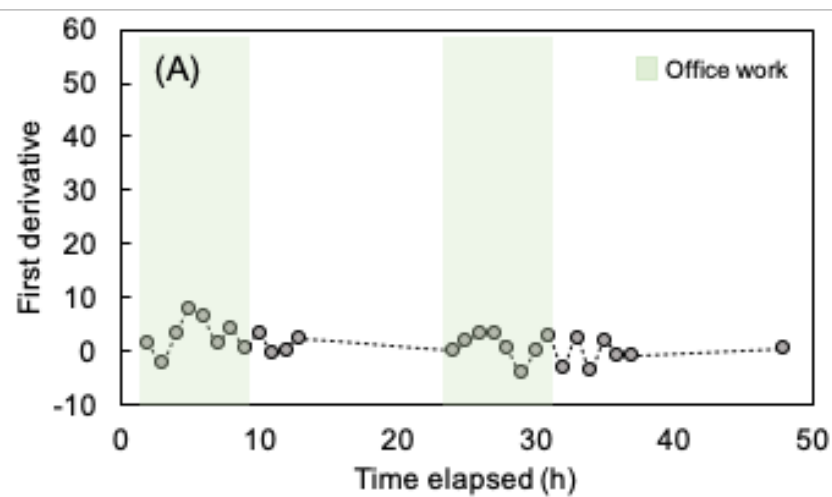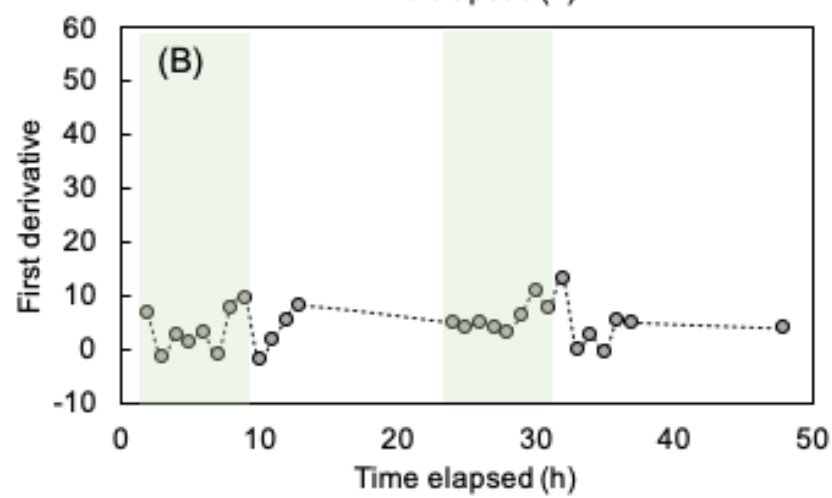

Supplement: S8 Fig — First derivative of sensor response over a 48 h period for pH sensors (A) and carbonyl sensors (B) deployed in an office. (PDF) [file pone.0258281.s008.pdf]

PCA - Biplot

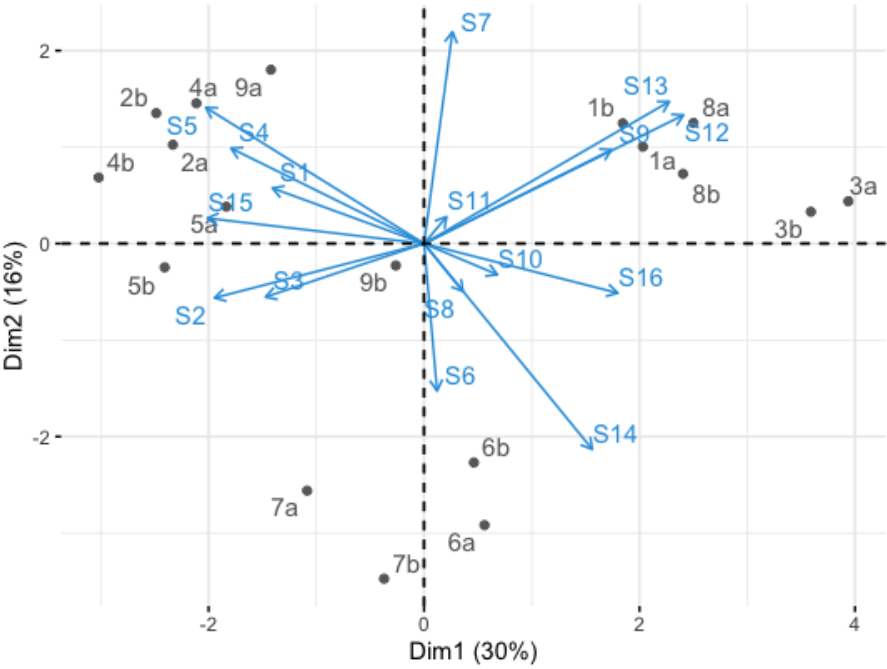

Supplement: S9 Fig — The arrows show the variables (sensors 1 to 16). The length of the arrows represent the contribution of each sensor to principal components 1 and 2. Two sensors were deployed in each household (labelled (a) and (b)). (PDF) [file pone.0258281.s009.pdf]

PCA - Biplot

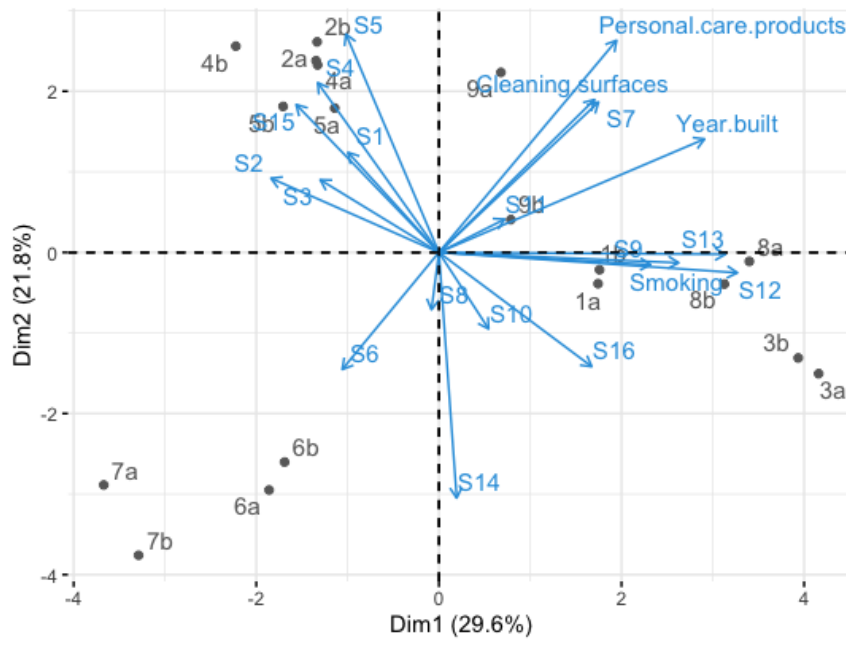

Supplement: S10 Fig — The arrows show the variables (sensors 1 to 16 and supporting information on cleaning, use of personal care products, smoking, year built). The length of the arrows represent the contribution of each sensor to principal components 1 and 2. Two sensors were deployed in each household (labelled (a) and (b)). (PDF) [file pone.0258281.s010.pdf]
