# Supplementary material for: Visualising household air pollution: Colorimetric sensor arrays for monitoring volatile organic compounds indoors
Source: PLoS One. 2021 Oct 6;16(10):e0258281. doi: 10.1371/journal.pone.0258281 (PMC8494322; doi:10.1371/journal.pone.0258281)
Supplement: S1 Table — (PDF) [file pone.0258281.s011.pdf]

Spot	Red (%RSD)	Green (%RSD)	Blue (%RSD)
S1	0.8	2.3	5.1
S2	2.2	2.2	1.9
S3	1.2	0.6	2.8
S4	1.5	4.5	2.7
S5	1.2	1.6	2.7
S6	0.8	1.2	2.8
S7	1.4	11.2	3.7
S8	2.9	43.8	5.9
S9	3.6	21.6	5.4
S10	3.1	4.2	1.9
S11	1.7	7.9	2.6
S12	0.9	1.1	26.4
S13	0.9	0.9	5.4
S14	2.5	4.5	5.9
S15	0.7	2.1	7.9
S16	0.2	2.2	4.5
