# Supplementary material for: Visualising household air pollution: Colorimetric sensor arrays for monitoring volatile organic compounds indoors
Source: PLoS One. 2021 Oct 6;16(10):e0258281. doi: 10.1371/journal.pone.0258281 (PMC8494322; doi:10.1371/journal.pone.0258281)
Supplement: S2 Table — (PDF) [file pone.0258281.s012.pdf]

Sample No.	Year built	Smoking	Spray cleaning	Personal care products
1	2003	No	Yes	Yes
2	1979	No	Yes	Yes
3	2014	Yes	Yes	Yes
4	1965	No	Yes	Yes
5	1977	No	Yes	Yes
6	1922	No	Yes	No
7	1928	No	No	No
8	2016	Yes	Yes	Yes
9	2008	Yes	Yes	Yes
