# Supplementary material for: Visualising household air pollution: Colorimetric sensor arrays for monitoring volatile organic compounds indoors
Source: PLoS One. 2021 Oct 6;16(10):e0258281. doi: 10.1371/journal.pone.0258281 (PMC8494322; doi:10.1371/journal.pone.0258281)
Supplement: S3 Table — (PDF) [file pone.0258281.s013.pdf]

Sample No.	pH sensors	Carbonyl sensors
1	39.74	223.00
2	81.12	257.36
3	48.21	379.65
4	81.38	246.56
5	90.58	277.08
6	61.21	258.12
7	60.72	244.96
8	51.05	319.45
9	77.65	241.58
